# Network-guided synthesis of antagonistic microbiota for suppressing maize stalk rot disease

**DOI:** 10.1093/ismejo/wrag243

**Published:** 2026-10-06

**Authors:** Da Li, Zishuo Qu, Wenzhe Feng, Xin Zhou, Lei Cai

**Affiliations:** State Key Laboratory of Microbial Diversity and Innovative Utilization, Institute of Microbiology, Chinese Academy of Sciences, Beijing 100101, P.R. China; College of Life Sciences, University of Chinese Academy of Sciences, Beijing 100049, P.R. China; State Key Laboratory of Microbial Diversity and Innovative Utilization, Institute of Microbiology, Chinese Academy of Sciences, Beijing 100101, P.R. China; College of Life Sciences, University of Chinese Academy of Sciences, Beijing 100049, P.R. China; State Key Laboratory of Microbial Diversity and Innovative Utilization, Institute of Microbiology, Chinese Academy of Sciences, Beijing 100101, P.R. China; State Key Laboratory of Microbial Diversity and Innovative Utilization, Institute of Microbiology, Chinese Academy of Sciences, Beijing 100101, P.R. China; State Key Laboratory of Microbial Diversity and Innovative Utilization, Institute of Microbiology, Chinese Academy of Sciences, Beijing 100101, P.R. China; College of Life Sciences, University of Chinese Academy of Sciences, Beijing 100049, P.R. China

**Keywords:** network analysis, microbial interactions, *Fusarium* stalk rot, synthetic community, biocontrol

## Abstract

Plant-associated microbiomes play a crucial role in sustaining host health and fitness, but how pathogen invasion reshapes microbial interaction networks and how to leverage this for developing effective biocontrol strategies remain poorly understood. This study investigated the maize microbiome under *Fusarium* stalk rot stress by integrating large-scale amplicon sequencing, network refinement, and experimental validation. To distinguish biological interactions from spurious associations driven by environmental and geographic factors, we applied a refined analytical framework to filter out abiotic correlations. The resulting interaction networks revealed that pathogen infection significantly increased network complexity and the proportion of potential negative interactions across all maize compartments except seeds. Specifically, potential negative interactions with *Fusarium* were significantly enriched in the rhizosphere microbiome, encompassing numerous uncultured taxa and known biocontrol genera (e.g. *Epicoccum*, *Burkholderia*, and *Nocardioides*). Representative strains from these taxa were subsequently isolated and assembled into a synthetic microbial community (SynCom). This refined network-derived SynCom significantly suppressed maize stalk rot caused by *Fusarium verticillioides* and *F. graminearum*. In non-sterilized soil, this SynCom exhibited significantly stronger disease-suppressive effects than either the randomly assembled SynCom or the SynCom derived from the unrefined network. Integrated transcriptomic and metabolomic analyses suggested that the SynCom confers resistance through the production of antifungal metabolites, including azelaic acid, and potentially through the induction of host defense responses. Collectively, our study provides meaningful insights into plant–microbe interaction under pathogen stress and suggests an analytical–experimental workflow that integrates computational ecology with experimental validation for developing biocontrol strategies against maize stalk rot.

## Introduction

Maize (*Zea mays* L.) is a globally important cereal crop, yet its production faces severe threats from several major pathogens. Among these, *Fusarium* stalk rot is a particularly destructive disease, responsible for substantial yield losses worldwide [1]. In China, this challenge is amplified by suboptimal agricultural management and climate change, leading to increased disease incidence and severity [2]. To mitigate its occurrence, conventional control has predominantly relied on the cultivation of resistant cultivars and the application of chemical pesticides. However, prolonged reliance on these approaches has fostered the emergence of pathogen resistance and resulted in environmental pollution [3], highlighting the pressing need for more sustainable crop protection solutions.

Diverse microbial communities comprising bacteria, fungi, archaea, protists, and viruses are intimately associated with plants and collectively form complex holobionts [4]. These microbial assemblages colonize distinct plant compartments, such as the rhizosphere, endosphere, stem, leaf, and seeds, collectively forming what is referred to as the plant “second genome.” Substantial evidence has established that the plant microbiome plays an indispensable role in facilitating host nutrient acquisition, enhancing pathogen resistance, and improving adaptability to environmental stresses [5–7]. For instance, previous research has demonstrated that maize stems specifically recruit *Gammaproteobacteria* to support biological nitrogen fixation [8]. More recent investigations have revealed that disease-resistant maize cultivars selectively enrich beneficial *Bacillus* species, which in turn activate the host’s metabolic defense pathways to counteract *Fusarium* infections [9]. These findings highlight that the strategic use of microbial resources is a promising and sustainable approach for enhancing plant disease resilience. However, a comprehensive understanding of the intricate interactions among microbes is a prerequisite for effectively harnessing their potential.

Microorganisms in natural ecosystems engage in intricate ecological interactions, encompassing both positive associations (e.g. mutualism and commensalism) that foster reciprocal benefits or unidirectional advantages, as well as negative relationships (e.g. competition and predation) that constrain the survival or reproduction of interacting partners [10]. Binary interaction networks, which model pairwise associations between microbial taxa, provide a useful framework for examining associations among microbial taxa [11, 12]. The wide application of high-throughput sequencing technologies has revolutionized this field by enabling efficient generation of large-scale microbial community profiling data, thereby facilitating the construction of microbial co-occurrence networks and supporting the inference of potential interactions within communities [13–15]. However, these network analyses, which infer associations based on correlations in species distribution and abundance data, inevitably capture both genuine biological interactions and spurious correlations arising from environmental factors [16]. This issue is particularly pronounced in large-scale microbial studies, where geographical distance and environmental heterogeneity exert substantial influences on microbial distributions [17–19]. In response, emerging methodologies seek to disentangle the ecological processes underlying the different types of associations in co-occurrence networks [20, 21]. For instance, a previously reported approach facilitates distinguishing associations driven by environmental selection and dispersal limitation, thereby refining the identification of potential biological interactions [20]. Despite these analytical advancements, experimental validation of these inferred interactions remains scarce.

Here, we asked whether separating associations attributable to environmental filtering and dispersal limitation from putative biotic interactions could improve the ability of large-scale microbiome networks to identify disease-suppressive microorganisms. To this end, we collected a total of 1410 samples from multiple maize compartments (including bulk soil, rhizosphere soil, roots, stems, and seeds) of both healthy and diseased plants across 33 sites in 23 provinces. Utilizing high-throughput sequencing of bacterial 16S rRNA gene and fungal internal transcribed spacer (ITS) regions, we constructed microbial co-occurrence networks to decipher community interaction patterns. By integrating multivariate statistics with network inference, we quantified the relative contributions of environmental selection, dispersal limitation, and biological interactions to microbial associations, with a focus on how pathogen invasion reshapes these interaction patterns. Finally, to validate the predictive capacity of the network, we isolated microbial taxa exhibiting potential negative interactions with *Fusarium* pathogens, constructed them into a synthetic microbial community (SynCom), and experimentally tested its efficacy in suppressing maize stalk rot.

## Materials and methods

### Sample collection and processing

A nationwide survey was conducted across 33 maize fields spanning 23.48–46.38°N and 100.27–126.57°E, with inter-site distances ranging from 40 to 3317 km [22]. These provinces collectively account for over 95% of China’s maize production areas. Fields under long-term continuous maize cultivation and exhibiting visible stalk-rot symptoms were selected; all plants were at the milk stage when sampled. Geographic coordinates, elevation, and stalk rot incidence and disease index were recorded for each plot. Each site was divided into five 10 m × 10 m sub-plots, and three healthy and three symptomatic plants were randomly chosen per sub-plot. From each plant, bulk soil, rhizosphere soil, root, stem, and seed samples were collected. Ears were placed in sterile bags; stems (nodes 2–4) were cut into three segments and pooled. Rhizosphere soil and roots were obtained after gentle shaking to remove loosely adhering soil, whereas bulk soil was sampled from inter-row zones (5–15 cm depth; three cores pooled). All specimens were kept on ice and transported under cold-chain conditions. Soil organic matter, total nitrogen, total phosphorus, total potassium (TK), available potassium, ammonium nitrogen, nitrate nitrogen, available phosphorus, available iron, pH, soil water content, and dissolved organic carbon were quantified using standard soil analytical protocols. Soil samples were collected from 33 sites with 5 replicates per site, totaling 165 samples. Mean annual temperature and precipitation were extracted from WorldClim v2.1 (www.worldclim.org). Soil types (black, brown, red) were identified using the China soil database (http://vdb3.soil.csdb.cn).

Root fragments were transferred to 50 ml tubes containing 20 ml sterile PBS and shaken at 120 rpm for 20 min to dislodge rhizosphere soil. Roots were then aseptically retrieved, and the suspension was centrifuged (8000 g, 5 min) to pellet rhizosphere soil. Surface sterilization of roots was performed as follows: 5% (v/v) NaOCl for 2 min, 75% ethanol for 1 min, and three rinses with sterile water; roots were quick-frozen in liquid nitrogen and pulverized. Stems and seeds underwent identical surface sterilization, rinsing, and were subsequently homogenized in 200 ml sterile water using a laboratory blender (Waring LB20ES). The homogenate was filtered through sterile gauze, and the filtrate was collected in a 50 ml tube. Endophytic microbial fractions were recovered by centrifugation (8000 g, 5 min) and stored at −80°C until DNA extraction.

### DNA extraction, sequencing, and data preprocessing

Total DNA was isolated using the FastDNA SPIN Kit for Soil (MP Biomedicals, USA) following the manufacturer’s instructions. DNA concentration and purity were quantified spectrophotometrically. For all 1410 samples, the V5–V6 region of the bacterial 16S rRNA gene and the fungal ITS2 region were amplified using primer pairs 799F/1115R (5′-AACMGGATTAGATACCCKG-3′/5′-AGGGTTGCGCTCGTTG-3′) and fITS7/ITS4 (5′-GTGARTCATCGARTCTTTG-3′/5′-TCCTCCGCTTATTGATATGC-3′), respectively, and sequenced on the Illumina NovaSeq platform. Sequence processing was performed in USEARCH v11.0 [23]: primer sequences were removed, and bases quality scores <30 were trimmed, paired-end reads were merged into full-length sequences, and ITS reads were additionally truncated to 200 bp and filtered (maximum expected error = 0.5). Zero-radius operational taxonomic units (ZOTUs) were inferred at 100% identity using the USEARCH–UNOISE3 pipeline [24]. Taxonomic assignments were obtained against SILVA (v138) [25] for bacteria and UNITE (v10.0) [26] for fungi.

### Data statistics and analysis

All statistical analyses were performed using the R environment (https://www.r-project.org/). To investigate the impact of pathogen infection on maize-associated microbial co-occurrence networks, we constructed co-occurrence networks using the igraph package [27]. Microbial taxa with an average relative abundance below 0.01% were filtered out. Pairwise associations were assessed using Spearman’s rank correlation, and only correlations with a coefficient |*ρ*| > 0.4 and a Benjamini–Hochberg adjusted *P* value <.05 were retained for network construction. Key topological properties of the resulting networks were calculated using igraph, including the number of nodes, number of positive and negative edges, total edges, average path length, network diameter, modularity, relative modularity, connectance, average degree, average clustering coefficient, number of clusters, betweenness centralization, and closeness centralization [11]. The network was randomized 999 times using the Erdős-Rényi random graph model, with the node and edge counts constrained to match those of the observed network. Key topological properties (average path length, clustering coefficient, modularity, diameter, and density) of the observed network were then compared against the distribution of these properties in the randomized networks. Canonical correspondence analysis (CCA) and variation partitioning analysis (VPA) were performed using the vegan package to quantify the individual and combined effects of environmental factors and geographic distance on microbial community composition [28].

To infer which assembly processes drive the associations between each species pair, we employed previously reported methods [20]. The abundance matrix was converted into a presence-absence matrix. For significantly associated ZOTU pairs, we recorded their co-occurrence patterns: co-presence or co-absence for aggregated (positively correlated) pairs, and mutually exclusive presence for segregated (negatively correlated) pairs. To test for spatial effects, we used multivariate analysis of variance (MANOVA) with plot coordinates (*X*, *Y*) as dependent variables and ZOTU co-occurrence pattern as the independent variable. To test for environmental effects, we performed MANOVA using the scores of the first two principal components from a principal component analysis (PCA) on soil parameters as dependent variables. Associations that were explainable by spatial distribution were attributed to dispersal limitation, whereas those explainable by environmental PCA axes were attributed to habitat filtering [29, 30]. Only associations not explained by spatial or environmental variables were classified as putative biotic interactions, with aggregated pairs considered potential positive interactions and segregated pairs considered potential negative interactions. Hereafter, correlations in the refined network are referred to as “potential interactions.” To assess the relative importance of environmental and spatial variables in mediating pairwise species associations, we performed a univariate analysis of variance for each species pair. The effect of each factor on association state (aggregated vs. segregated) was evaluated using *F* values and the magnitude of explanatory power was quantified by eta-squared (*η^2^*). Geographic coordinates (UTM_X, UTM_Y) were also included to account for potential spatial structuring in the species associations. To assess whether phylogenetic distances differed among inferred community assembly processes, we compared the phylogenetic distances between ZOTU pairs associated with each process. A phylogenetic tree of taxa in the network was constructed using maximum likelihood methods. Phylogenetic trees were constructed separately within individual kingdoms and across kingdoms. Subsequently, using the ape package, the phylogenetic distances in calibrated trees were calculated for all pairs of aggregated or segregated ZOTUs, with branch lengths representing evolutionary time, indicated in millions of years.

To investigate taxa potentially involved in competition with *Fusarium*, all taxa showing potential negative interactions with ZOTUs assigned to the genus *Fusarium* were extracted, and their abundance was calculated across different niches. We compared the distribution of all *Fusarium*-annotated ZOTUs across different plant compartments and constructed a phylogenetic tree using the maximum likelihood method based on the ITS sequences to infer their potential taxonomic positions. All rhizosphere taxa negatively correlated with *Fusarium* were subsequently visualized using the igraph package. Given the compositional nature of amplicon data, we also constructed microbial association networks in the diseased rhizosphere using sparse inverse covariance estimation for ecological association and statistical inference (SPIEC-EASI) with the Meinshausen–Bühlmann neighborhood selection method. Additionally, to identify taxa negatively associated with *Fusarium* ZOTUs, individual networks were inferred separately for each of the 33 sampling sites. To assess the impact of removing environmental selection and dispersal limitation on network data mining, random forest analysis was performed on *Fusarium*-negatively correlated taxa before and after removal. Specifically, model construction was based on the rfPermute package in R [31], using negatively correlated taxa to regress against variations in *Fusarium* abundance. Key parameter settings were as follows: the number of decision trees (ntree) was set to 500 to ensure stable model prediction performance; to evaluate the statistical significance of feature importance, the number of permutations (nrep) was set to 1000 to obtain robust *P* values. To investigate the influence of geographic and environmental distances on networks before and after the removal of associations attributed to environmental selection and dispersal limitation, distance–decay relationship analysis was conducted. Based on plot latitude and longitude coordinates, geographic distances between plots were calculated using the geosphere package in R. Euclidean distances were calculated based on soil factor and climate factor data to serve as metrics for soil environmental distance and climate distance, respectively. All schematic figures were created with biorender (https://biorender.com/).

### Isolation of bacteria and fungi from maize rhizosphere

Microbes were isolated from rhizosphere soil suspensions. Briefly, 3 g of soil was suspended in 30 ml of sterile solution and shaken at 180 rpm for 30 min. The resulting suspension was serially diluted from 10^−3^ to 10^−6^, and 100 μl aliquots of appropriate dilutions were spread onto Luria–Bertani agar (LB), tryptic soy agar (TSA), and potato dextrose agar (PDA) plates for bacterial and fungal isolation. Distinct colonies were selected, and their 16S rRNA gene or ITS regions were amplified and sequenced using universal primers. Taxonomic classification was assigned using the NCBI pipeline, and a maximum-likelihood phylogenetic tree was constructed with FastTree 2.1.11 [32]. To validate the disease-resistance roles of the taxa identified through network mining, candidate isolates were ranked according to marker-region sequence identity, and the highest-identity cultured representative available for each target ZOTU was selected. The refined network identified six microbial strains, including *Burkholderia* sp. LDB271, *Pseudarthrobacter* sp. LDB139, *Arthrobacter* sp. LDB377, *Nocardioides* sp. B267, *Cladosporium* sp. ph 14, and *Epicoccum* sp. F470. The antagonistic activities of these six isolates against the pathogenic fungus *Fusarium verticillioides* LD38 were subsequently evaluated on agar plates [6].

### Construction of SynCom

To systematically evaluate the overall pathogen-resistance capacity of the selected strains, we constructed a SynCom. The SynCom consisted of bacterial and fungal cells in a 1:1 ratio. Bacterial strains were cultured in tryptic soy broth (TSB) medium, harvested by centrifugation (4000 rpm, 10 min) at OD_6_₀₀ = 1, and resuspended in phosphate-buffered saline (PBS) to a density of 1 × 10^8^ CFU g^−1^ soil. Fungal conidial suspensions were prepared in PBS and adjusted to 1 × 10^8^ spores g^−1^ soil, as determined using a hemocytometer. Maize seeds were surface-sterilized, soaked for 24 h, pre-germinated in a moist chamber for 48 h, and transplanted into non-sterile soils. Soil samples from 33 nationwide sites were homogenized in equal weight proportions and mixed with vermiculite at a 2:1 (v/v) ratio. Plants were grown under a 16/8 h light/dark cycle at 25°C. After 7 days, a six-strain SynCom mixture was applied to the rhizosphere. Simultaneously with SynCom application, soils were inoculated with *F. verticillioides* LD38 and *F. graminearum* LD190 at a final concentration of 1 × 10^7^ spores g^−1^ soil [33]. Disease incidence, disease index, fresh weight, plant height, and root length were recorded 15 days post-inoculation. Each treatment included 25 plants (5 per pot), with two experimental replicates.

To validate the efficacy of the network refinement approach, we constructed three SynComs to assess the disease-suppressive potential of taxa identified through network mining. These included: (i) a random SynCom assembled from randomly selected isolates; (ii) a SynCom composed of taxa obtained from unrefined network mining; and (iii) a SynCom formed by taxa from refined network mining. To ensure community consistency, each SynCom comprised four bacterial and two fungal strains. Strains for random communities were selected using a random number generator. For communities derived from unrefined networks, representative strains were identified as the isolates exhibiting the highest sequence similarity to the amplicon sequences. Subsequently, bacteria and fungi meeting this criterion were randomly chosen using a random number generator. All control groups received an equal volume of PBS along with the pathogen at the same concentration as the experimental groups. The antagonistic activity of each consortium against the pathogen *F. verticillioides* LD38 was evaluated using a plate confrontation assay. Furthermore, individual members from the SynCom were also inoculated separately to determine their respective contributions to plant disease resistance.

### Antifungal assay of metabolites from individual strains and the SynCom

To evaluate the effect of metabolites from individual strains or the SynCom on the pathogen, complementary in vitro assays were conducted. For individual strains, bacteria or fungi were cultured in tryptic soy broth (TSB) or potato dextrose broth (PDB) medium, respectively, at 28°C with shaking at 180 rpm for 7 days. For the SynCom, bacterial and fungal strains were adjusted to a uniform cell density before co-inoculation into PDB medium and incubated under the same conditions for 7 days. After fermentation, each culture was filtered, centrifuged, and the supernatant was extracted three times with ethyl acetate. The combined organic phases were rotary-evaporated to dryness to obtain a crude extract. The residue was dissolved in 100 μl of methanol and incorporated into molten PDA medium at a final concentration of 256 μg ml^−1^. Once solidified, 6 mm wells were punched in the agar and inoculated with *F. verticillioides* LD38. Inhibitory activity was assessed by measuring the radius of mycelial growth after four days of incubation.

### Assessment of SynCom rhizosphere colonization

To evaluate the rhizosphere colonization capacity of the SynCom, we collected maize rhizosphere soil samples at 10, 20, and 30 days post-inoculation (dpi) from plants inoculated with a randomly assembled SynCom, an unrefined-network SynCom, or a refined-network SynCom. Total genomic DNA was extracted from all 36 rhizosphere samples and used to amplify the bacterial 16S rRNA gene (27F: 5′-AGRGTTYGATYMTGGCTCAG-3′; 1492R: 5′-RGYTACCTTGTTACGACTT-3′) and the fungal ITS region (ITS9: 5′-TACACACCGCCCGTCG-3′; ITS4: 5′-CCTSCSCTTANTDATATGC-3′). Amplicon libraries were constructed and sequenced on the PacBio Sequel platform to generate high-fidelity reads. Raw reads were quality-filtered, demultiplexed, and primer-trimmed using VSEARCH. Dereplication was performed prior to ZOTU inference via the UNOISE3 algorithm. Potential chimeras were identified and removed by comparison against the RDP reference database. Clean ZOTUs were then mapped back to the high-quality reads to generate a feature table. Finally, representative ZOTU sequences were subjected to BLASTn analysis against a custom database comprising the sequences of the inoculated strains to track the colonization and persistence of the inoculated strains in the rhizosphere.

### Metabolomic analysis of individual strains and the SynCom

Crude extracts obtained from fermentation broths were subjected to metabolomic analysis. Fermentation broths were extracted with an equal volume of ethyl acetate, followed by LC–MS/MS analysis on the UHPLC system under the following conditions: flow rate, 0.4 ml min^−1^; positive ion mode: mobile phase A, aqueous solution containing 0.1% formic acid, mobile phase B, acetonitrile; negative ion mode: mobile phase A, 5 mM ammonium acetate, mobile phase B, acetonitrile; column, Waters ACQUITY UPLC BEH C18 (130 Å, 1.7 μm, 2.1 × 100 mm); column temperature, 35°C; total run time, 25 min. Metabolites were annotated using the Human Metabolome Database (https://hmdb.ca/) and the Natural Products Atlas (https://www.npatlas.org/). Compounds were compared to identify metabolites shared between individual strains and the synthetic community. A random forest model was applied to evaluate the potential contribution of the top 50 relatively abundant compounds to the in vitro antifungal activity. For compounds for which authentic standards were available, the residue was dissolved in 100 μl methanol and incorporated into molten PDA medium at a final concentration of 128 μg ml^−1^. After solidification, 6 mm wells were punched and inoculated with *F. verticillioides* LD38. Inhibitory activity was assessed by measuring the mycelial growth radius after four days of incubation. To investigate rhizosphere concentrations of metabolites, both untargeted and targeted metabolomics analyses were performed on rhizosphere soils with and without SynCom inoculation. Briefly, 1 g freeze-dried soil sample was extracted with ethyl acetate. The crude extract was collected and redissolved in 4 ml methanol. After centrifugation, the supernatant was transferred to a sample vial for UPLC-Q-TOF-MS injection. Untargeted metabolomic profiling was conducted using UHPLC. Targeted metabolite quantification was achieved via an external calibration curve constructed using an authentic standard, where ion peak area was plotted against standard concentration. The final concentration of target metabolite in soil was calculated by taking into account the extraction volume, injection aliquot, and soil sample weight.

### Transcriptomic analysis of maize inoculated with individual strains and the SynCom

Maize seeds were surface-sterilized, soaked for 24 h, pre-germinated in a moist chamber for 48 h, and transplanted into sterile soil. Plants were grown under a 16/8 h light/dark cycle at 25°C. After 7 days, individual strains or the SynComs were inoculated at a density adjusted to 1 × 10^8^ CFU g^−1^ soil. The SynComs consisted of three groups: random SynCom, SynCom derived from unrefined microbial networks, and SynCom derived from refined microbial networks. Leaf and root samples were carefully collected 48 h post-inoculation. Total RNA was extracted from root and leaf tissues of maize samples, and mRNA was enriched to construct a transcriptomic library; high-throughput sequencing was performed on the Illumina platform with PE150 read length to generate raw sequencing data. Raw RNA-seq reads were quality-assessed using FastQC and summarized with MultiQC [34]. Clean reads were then aligned to the maize reference genome (Zm–B73–REFERENCE–NAM–5.0, GCF_902167145.1) using HISAT2 in a splice-aware mode, with splice sites and exons extracted from the annotation file [35]. Gene-level quantification was performed using Salmon in mapping-based mode with GC bias correction [36], employing a transcriptome index built from the reference genome and annotation. A read-count matrix was subsequently generated from the Salmon quantifications for downstream differential expression analysis. Differentially expressed genes were identified using edgeR with a significance threshold of an FDR-adjusted *P* < .05 and an absolute log_2_(fold change) > 1. Gene ontology (GO) enrichment analysis was subsequently performed on the upregulated genes from the strain or community inoculation groups.

## Results

### Refined network analysis reveals increased potential biotic interactions under pathogen stress

To investigate the impacts of pathogen infection on microbial community interactions in maize, we constructed microbial co-occurrence networks (Supplementary Fig. S1; Supplementary Table S1). These networks exhibited significant structural differences from their randomized counterparts (Supplementary Table S2), indicating non-random assembly patterns. Considering that species coexistence is governed by a combination of environmental selection, dispersal limitation, and biological interactions [30], we sought to disentangle these forces to identify putative biotic interactions under pathogen stress. CCA and VPA revealed that both environmental factors and geographical distance significantly shaped microbial community composition and structure (*P* < .05), particularly in below-ground compartments (Supplementary Fig. S2). This finding underscores the necessity of accounting for these influences before inferring putative biological interactions within maize microbial communities under pathogen stress.

To distinguish network connections attributable to environmental selection and dispersal limitation from potential biological interactions, we employed the analytical framework [20]. This refinement revealed that over 50% of the associations in the original co-occurrence networks could be attributed to environmental selection and dispersal limitation collectively (Fig. 1a; Supplementary Tables S3–S5). Among these, mean annual precipitation, mean annual temperature, pH, and spatial distance were shown to be the key factors influencing microbial associations (Supplementary Fig. S3; Supplementary Table S4). Pathogen infection increased the proportion of potential biological interactions across all plant-associated compartments except seeds (Fig. 1a). To further assess the role of phylogenetic relationships in structuring these co-occurrence patterns, we calculated phylogenetic distances between taxon pairs exhibiting statistically significant associations. Our results showed that potential negative interactions occurred predominantly between phylogenetically distant taxa, whereas positive interactions and environmentally filtered associations were more frequent among closely related taxa (Fig. 1b; *P* < .05). This pattern was highly consistent with observations from the combined bacterial–fungal phylogenetic tree (Supplementary Fig. S4a). Under the stress of pathogen infection, taxa exhibiting potential positive interactions within the microbial network demonstrated closer phylogenetic distance (Supplementary Fig. S4b; *P* < .05).

**Figure 1:**
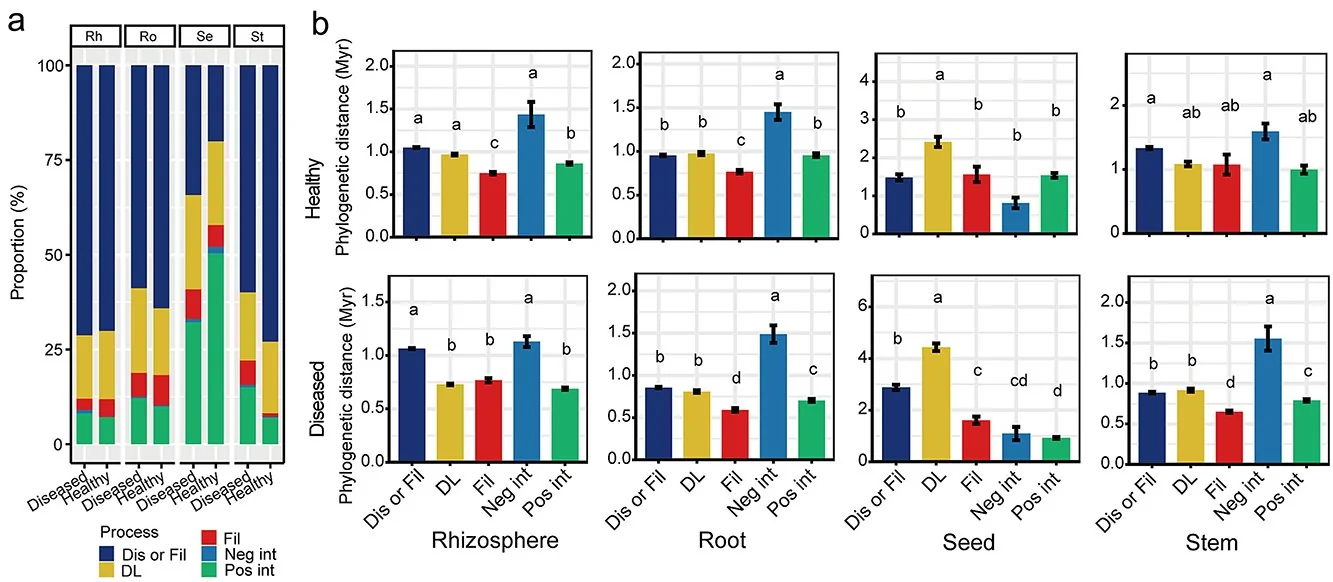
Driving forces of microbial co-occurrence patterns in maize under *Fusarium* stalk rot stress. (a) Proportions of connections in the co-occurrence network attributed to environmental filtering, dispersal limitation and potential biological interactions. (b) Phylogenetic distances between taxa linked by co-occurrence relationships governed by environmental filtering, dispersal limitation, and biological interactions. Phylogenetic trees for bacteria and fungi were constructed separately using the maximum-likelihood method. Rh, rhizosphere; Ro, root; St, stem; Se, seed; Dis or Fil: dispersal limitation or filtering, DL: dispersal limitation, Fil: filtering, Neg int: negative interaction, Pos int: positive interaction. Different letters indicate statistically significant differences between groups (*P* < .05).

### Pathogen infection enhanced network complexity in maize-associated microbial community

Following the removal of connections attributed to environmental selection and dispersal limitation, we constructed potential interaction networks that exhibited significant structural differences from their randomized networks (Supplementary Tables S6 and S7). Comparative analysis of the rhizosphere, root, and stem compartments revealed that although diseased maize networks often contained fewer nodes, they consistently exhibited more edges and greater overall network complexity compared to healthy plants (Fig. 2; Supplementary Fig. S5). Specifically, these diseased compartments displayed a higher proportion of potential negative interactions (Fig. 2; Supplementary Fig. S5). Conversely, the seed-associated networks from diseased maize contained more nodes but fewer edges than those from healthy seeds. Collectively, these findings indicate that pathogen infection substantially enhances the structural complexity of maize-associated microbial networks.

**Figure 2:**
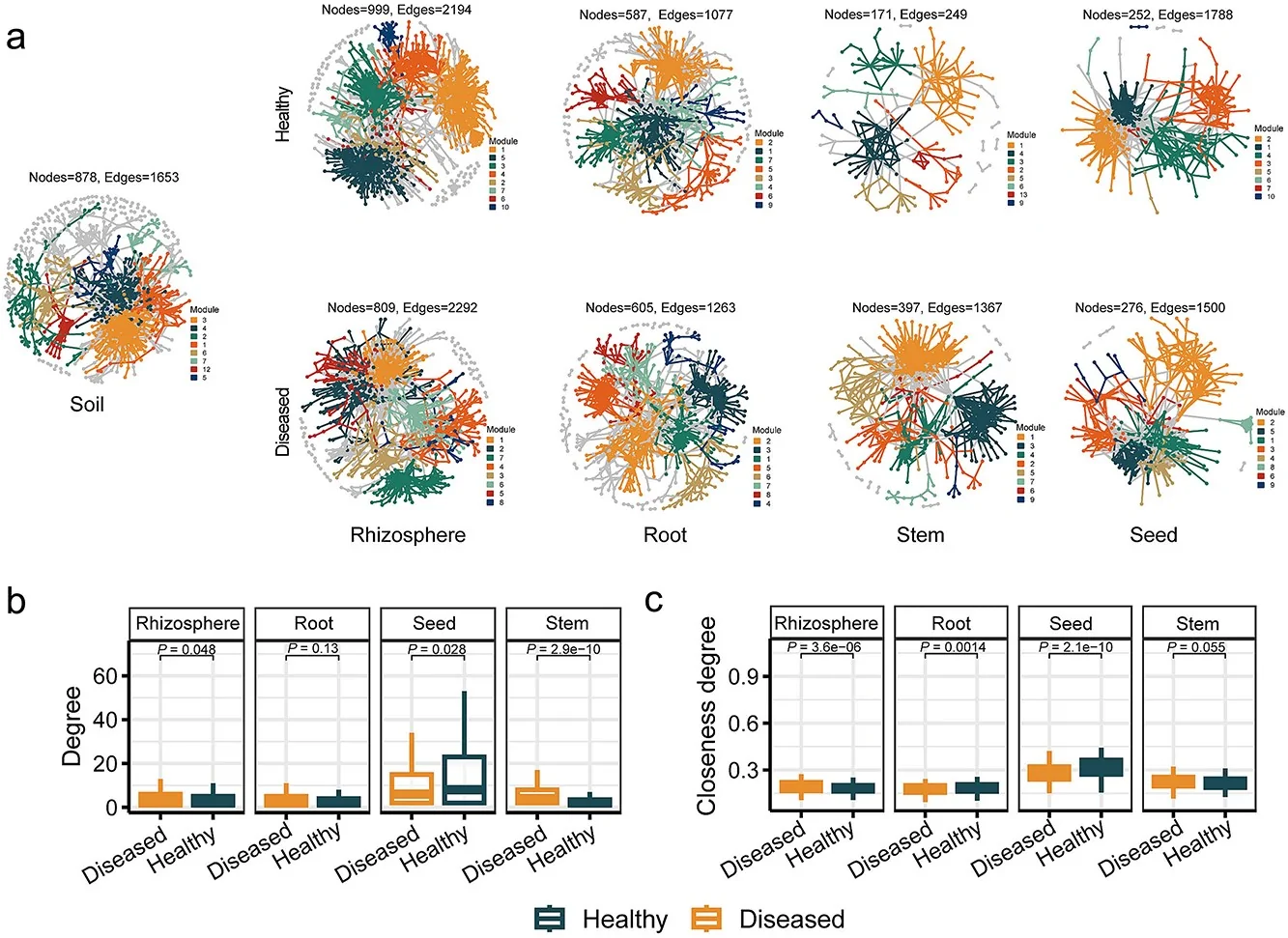
Effects of *Fusarium* stalk rot infection on potential bacterial–fungal interaction networks in maize. (a) Visualization of microbial networks across distinct maize niches under pathogen stress. The eight largest modules are highlighted in color, whereas the remaining modules are depicted in gray. (b) Topological properties of microbial networks across maize niches under pathogen stress.

To investigate kingdom-specific interactions, we constructed separate bacterial and fungal co-occurrence networks (Supplementary Fig. S6). After applying the same refinement procedure, we identified contrasting interaction patterns between kingdoms in response to pathogen infection (Supplementary Fig. S7). Pathogen infection significantly enhanced both node connectivity and potential positive interactions within bacterial networks. In contrast, fungal networks displayed reduced node connectivity, diminished positive interactions, and an increased prevalence of potential negative interactions under disease conditions (Supplementary Fig. S7).

### Pathogen infection enriched *Fusarium*-negative taxa in maize microbiome

To identify microbial taxa interacting with *Fusarium* pathogens and potential biocontrol taxa, we extracted all taxa associated with *Fusarium* ZOTUs from the co-occurrence networks. Compared to healthy tissues, over 66% of *Fusarium*-affiliated ZOTUs showed significantly higher relative abundance in diseased maize tissues (Supplementary Fig. S8; *P* < .05) and clustered phylogenetically with known pathogens responsible for maize root, stalk, and ear rots (Supplementary Fig. S9). After refining the networks by removing associations driven by environmental factors and geographical distance to ensure robust prediction of biological interactions, we observed that pathogen infection significantly increased the number of taxa exhibiting potential negative interactions with *Fusarium* species compared to healthy maize plants, with this effect being particularly pronounced within the rhizosphere microbial community (Fig. 3a). Subsequent characterization of these negatively correlated taxa identified numerous uncultured and undescribed microbial species (Fig. 3b; Supplementary Table S8), alongside several known biocontrol genera including *Epicoccum*, *Burkholderia*, *Nocardioides*, *Pseudarthrobacter*, *Mortierella*, *Flavisolibacter*, and *Vishniacozyma* (Fig. 3b). We constructed separate co-occurrence networks for each of the 33 individual sampling sites and extracted taxa that were negatively correlated with *Fusarium* ZOTUs. The overlap between these site-specific taxa and those identified from the refined network ranged from 10.5% to 81.6% (Supplementary Fig. S10; Supplementary Tables S9 and S10), whereas the overlap with the network constructed using SPIEC-EASI was 15.8% (Supplementary Fig. S11, Supplementary Table S11). We further validated the ecological relevance of these refined correlations through random forest analysis, which demonstrated that the negative taxa identified after network refinement exhibited significantly stronger explanatory power for *Fusarium* abundance variation compared to those from the unrefined network (Fig. 4a and b; *P* < .05). Furthermore, the distance–decay relationship analysis revealed that the taxa exhibiting potential negative interactions with *Fusarium* after refinement were less influenced by environmental selection and dispersal limitation (Fig. 4c–e), and this trend was more pronounced in stem compartments (Supplementary Fig. S12). Collectively, these findings indicate that pathogen infection enhances potential competitive or antagonistic interactions within the architecture of maize microbiome interaction networks.

**Figure 3:**
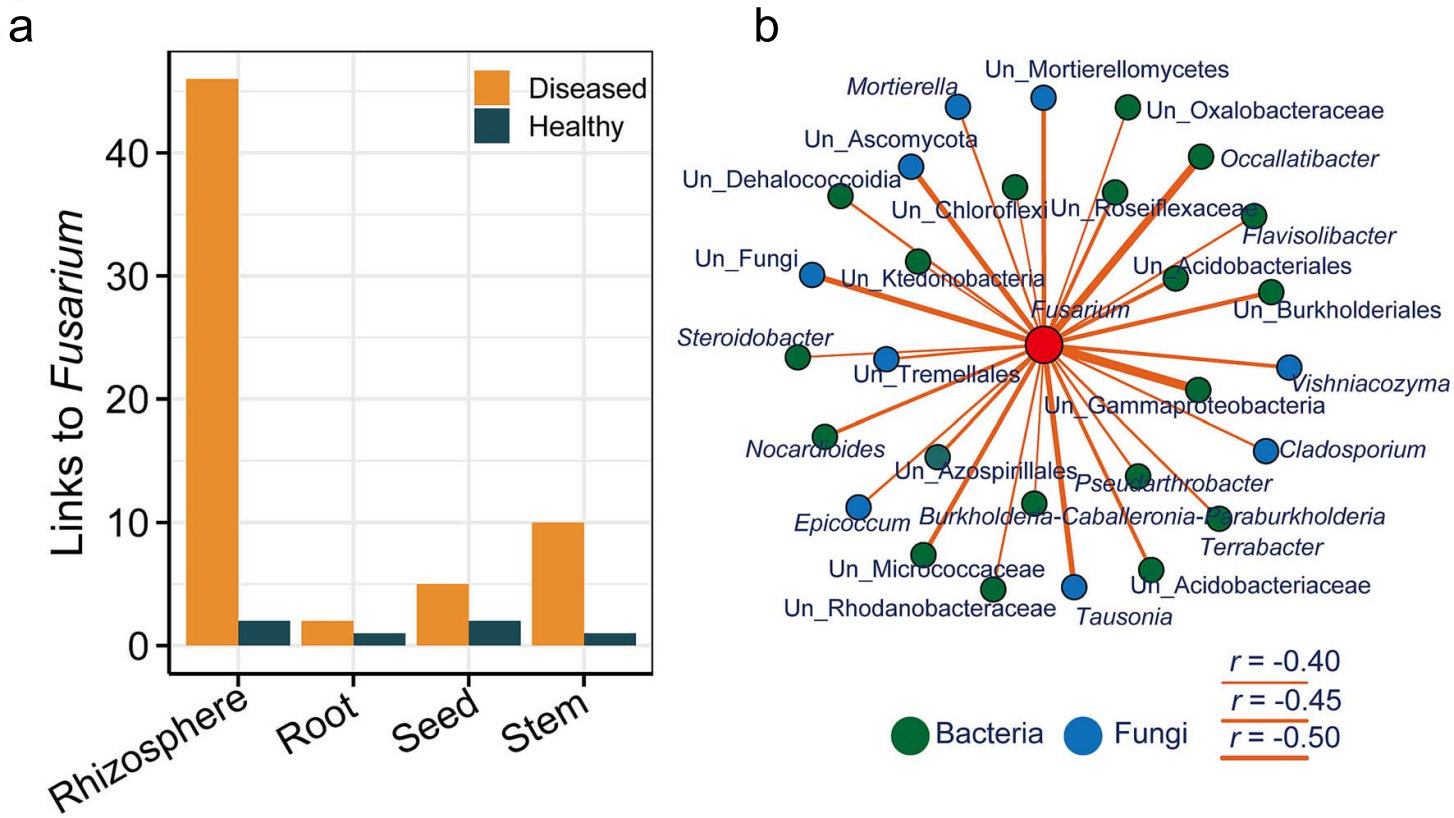
Potential interactions between the maize microbiome and *Fusarium* under stalk rot stress. (a) Numbers of negatively correlated taxa with *Fusarium* in microbial potential interaction networks across distinct maize niches under pathogen stress. (b) Microbial taxa negatively correlated with *Fusarium* in the rhizosphere of diseased maize. The central node represents the *Fusarium*-affiliated ZOTU, and the surrounding nodes represent bacterial and fungal taxa. Edge thickness is proportional to the absolute value of Spearman's correlation coefficient (*P* < .05).

**Figure 4:**
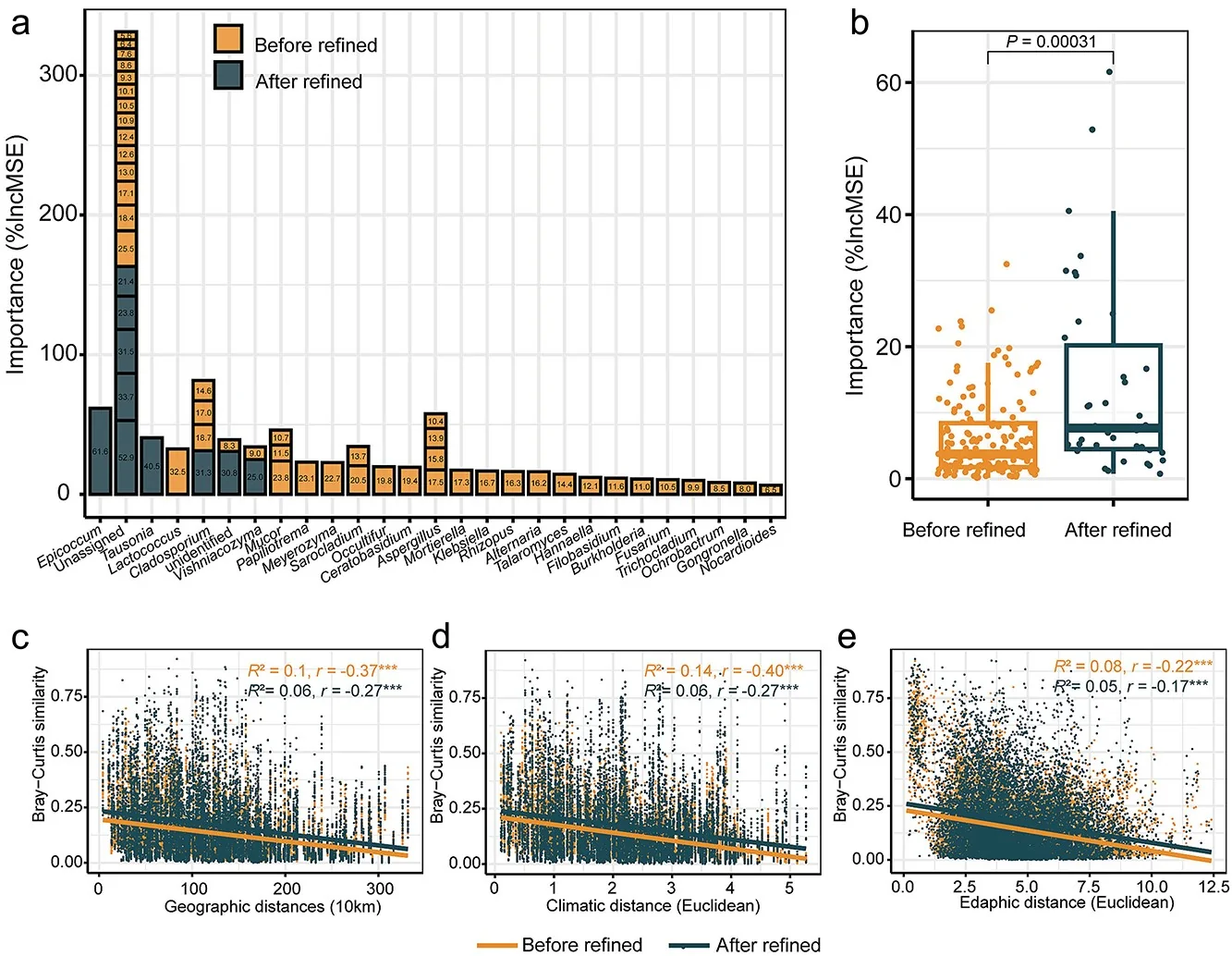
Effects of network refinement on microbial taxa negatively correlated with *Fusarium*. (a) Comparison of *Fusarium*-negatively correlated taxa identified from potential interaction networks before and after removing associations attributable to environmental and geographic variation. Only taxa with significant differences (*P* < .05) in random forest models are shown. (b) Importance of *Fusarium-*negatively correlated taxa for *Fusarium* relative abundance variation before and after removing taxa associated with environmental and/or geographic variation. (c–e) Distance–decay curves showing Bray–Curtis similarity against geographic distances (c), climatic distances (d), and edaphic distances (e). Asterisks denote statistical significance: ^***^P < .001.

### Synthetic communities effectively enhance maize resistance against stalk rot

To validate the network-predicted biocontrol potential and assess the efficacy of these taxa against maize stalk rot, we isolated and cultured rhizosphere microorganisms from maize plants (Supplementary Table S12). We matched ZOTUs derived from amplicon sequencing against full-length 16S rRNA gene/ITS sequences of cultured isolates and recovered six strains representing the rhizosphere taxa prioritized by the refined network, including four bacterial and two fungal isolates: *Burkholderia* sp. LDB271, *Pseudarthrobacter* sp. LDB139, *Arthrobacter* sp. LDB377, *Nocardioides* sp. B267, *Cladosporium* sp. ph 14, and *Epicoccum* sp. F470. The marker gene sequences of these isolates shared 97.7%–100% identity with their corresponding network ZOTUs across the sequenced amplicon regions (Fig. 5a; Supplementary Table S13). Accordingly, these isolates were regarded as culturable representatives of the network-prioritized taxa. All six network-identified strains were subsequently assembled into a SynCom to evaluate its disease-suppressive potential. Under non-sterile soil conditions, the SynCom significantly reduced the disease index of maize stalk rot caused by two major *Fusarium* pathogens, *F. verticillioides* and *F. graminearum* (Fig. 5b; Supplementary Fig. S13, *P* < .05). To investigate the disease-suppressive potential of the refined network-derived SynCom, we assembled two control communities: a random SynCom and a SynCom based on the unrefined co-occurrence network. Inoculation assays showed that the SynCom derived from the refined network provided significantly stronger control efficacy against maize stalk rot than either the random SynCom or the unrefined network SynCom (Fig. 5c; *P* < .05). The individual strain inoculation assays revealed that strains selected from the refined network exhibited a higher proportion of disease-suppressive activity and greater inhibitory potency against the pathogen, relative to strains from the random set or the unrefined network (Supplementary Figs. S14 and S15). Amplicon sequencing revealed distinct colonization capacities among synthetic communities. Most members of the refined SynCom remained detectable in the rhizosphere across the sampling period, with the exception of *Pseudarthrobacter* sp. LDB139. In contrast, only four strains from the unrefined network and three from the random SynCom were detected in the rhizosphere (Supplementary Fig. S16).

**Figure 5:**
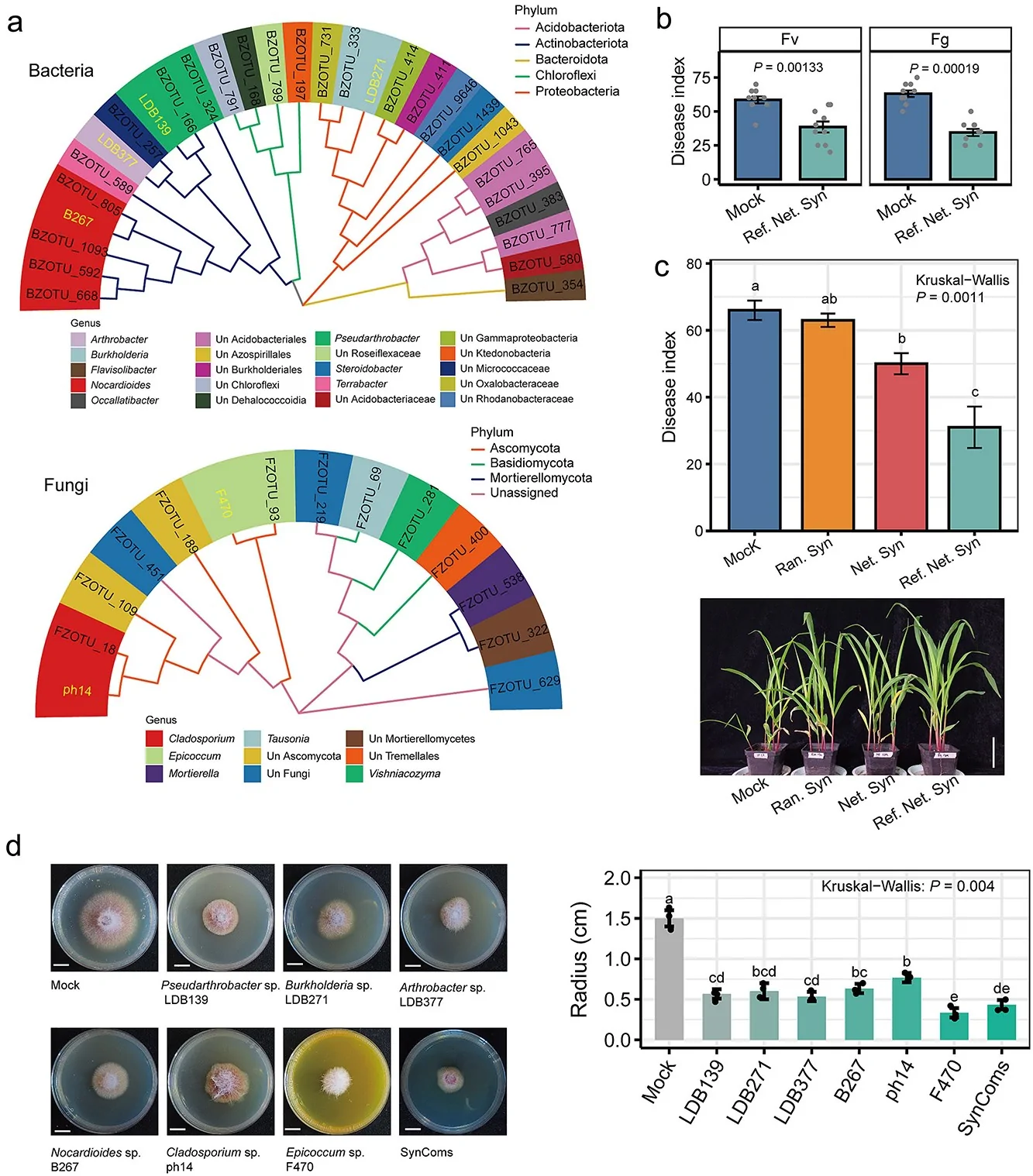
Effects of synthetic communities constructed from microbial network-associated taxa on maize stalk rot. (a) Phylogenetic tree of microbial network-identified negatively correlated taxa and isolated strains. The tree was constructed using the maximum likelihood method. Branch colors represent different bacterial phyla, and outer ring colors indicate genera. (b) Effect of SynCom on disease index of maize stalk rot (*n* = 10). Fv: *F. verticillioides* LD38; Fg: *F. graminearum* LD190. (c) Effects of the random community, the unrefined network SynCom, and the refined network SynCom on the disease index of maize stalk rot (scale bar = 10 cm, *n* = 5). (d) Effects of metabolites from a single strain and SynCom on the growth of *F. verticillioides* LD38 under in vitro conditions (scale bar = 1 cm, *n* = 3). Different lowercase letters indicate significant differences between treatments (Kruskal–Wallis test, *P* < .05).

To investigate the disease-suppression mechanisms of the refined network-derived taxa, we extracted metabolites from both individual strains and the SynCom. All extracts significantly inhibited the growth of *F. verticillioides* LD38. Among them, *Epicoccum* sp. F470 and the SynCom exhibited the strongest antifungal activity (Fig. 5d; *P* < .05). Further metabolomic analysis revealed that over 60% of the compounds identified in individual strains were stably present in the SynCom (Supplementary Fig. S17). Random forest analysis indicated that, among the top 50 most abundant compounds, 20 compounds were closely associated with the pathogen inhibition capacity (Fig. 6a; Supplementary Table S14; *P* < .05). Among the obtained compounds, boholamide A, fragin, lysophosphatidylcholine (lysoPC), and azelaic acid significantly inhibited the growth of *Fusarium* (Fig. 6b and c; *P* < .05). Untargeted metabolomics showed that boholamide A, lysoPC, and azelaic acid accumulated significantly upon SynCom inoculation compared with the mock control (Fig. 6d; *P* < .001). Targeted metabolomics verified a significantly higher absolute rhizosphere concentration of azelaic acid in the inoculated SynCom (Fig. 6e and f; *P* < .001), while other metabolites fell below the limit of detection. Transcriptomic data showed that inoculation with either individual strains or the SynComs significantly altered gene expression in maize roots and leaves (Fig. 7a; Supplementary Figs. S18 and S19; Supplementary Table S15). GO enrichment analysis indicated that inoculation with the SynCom activated putative salicylic acid-mediated immune pathways and defense responses in the roots, but not in the leaves (Fig. 7b; Supplementary Fig. S20; *P* < .05). We did not observe induction of putative host immune-related genes upon inoculation with the random SynCom. Inoculation with the SynCom derived from the unrefined network also altered defense-related gene expression in leaves, but did not reproduce the prominent defense-associated enrichment pattern observed in roots following inoculation with the refined-network SynCom (Supplementary Fig. S18; Supplementary Table S16). These results indicate that the superior disease suppression of the refined-network SynCom demonstrates that removing environmentally and geographically structured associations enriched the candidate pool for microorganisms with experimentally detectable disease-suppressive activity, supporting the functional relevance of network-predicted taxa with potential negative interactions with *Fusarium*, which may enhance maize resistance to stalk rot through antifungal metabolite production and the potential priming of host defense responses (Fig. 7c).

**Figure 6:**
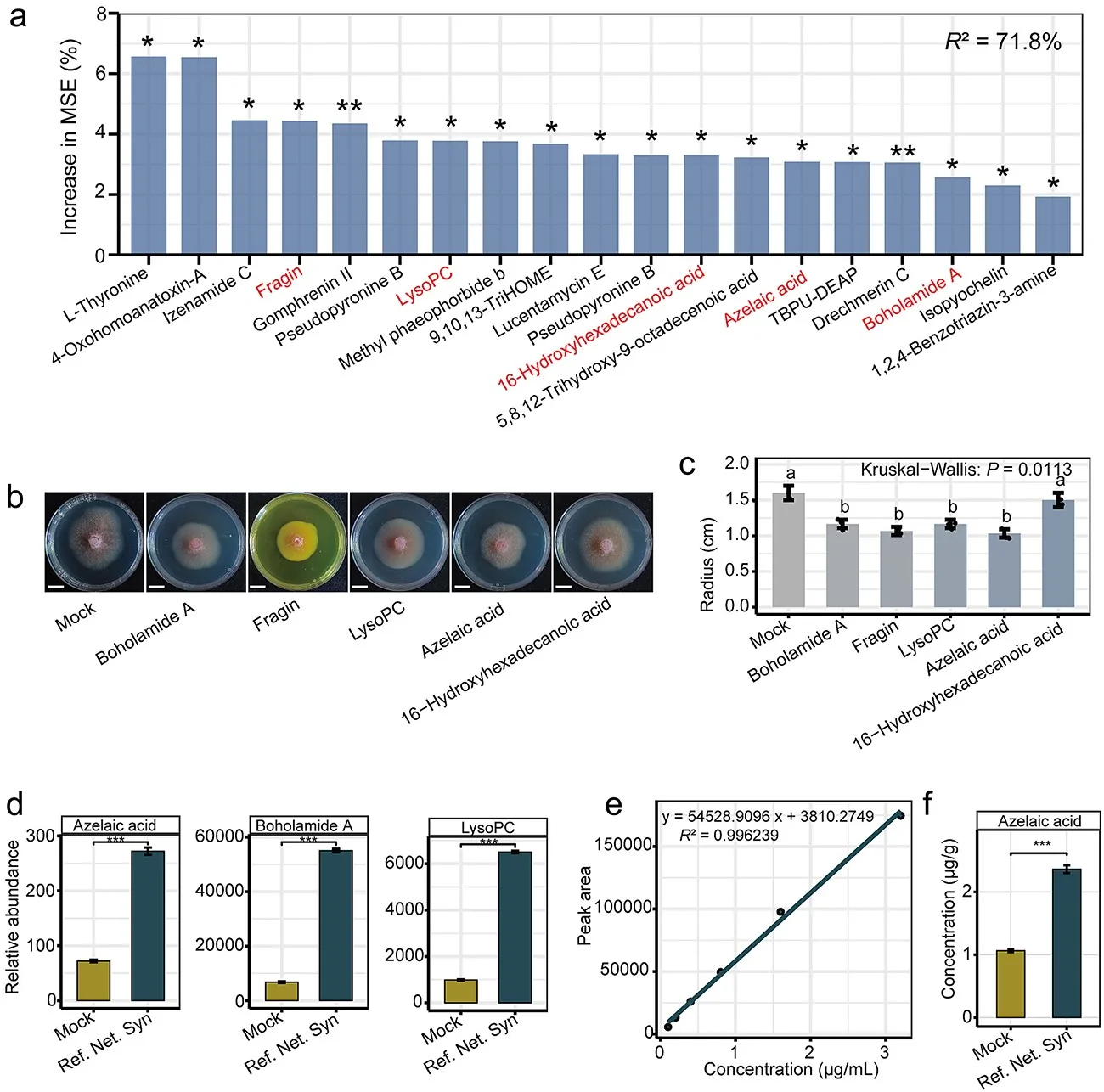
Effects of metabolites produced by individual strains and the SynCom on *F. verticillioides* LD38 inhibition. (a) Random forest analysis of the 50 most abundant compounds for predicting inhibitory activity against *F. verticillioides* LD38. (b) Inhibitory effects of selected compounds on *F. verticillioides* LD38 (scale bar = 1 cm). (c) Inhibition capacity of the selected compounds against *F. verticillioides* LD38 (*n* = 3). (d) Relative abundance of three annotated metabolites (azelaic acid, boholamide A, and lysoPC) obtained from untargeted metabolomics analysis in mock and refined SynCom treatments. (e) UPLC-Q-TOF-MS external calibration curve for targeted quantification of azelaic acid. Peak area was plotted against standard concentration. (f) Absolute concentration of azelaic acid in rhizosphere soil determined by targeted quantification. Bars represent mean values, and error bars indicate standard error of the mean. Asterisks denote statistical significance (^*^P < .05; ^**^P < .01; ^***^P < .001). Different lowercase letters indicate significant differences between treatments (Kruskal–Wallis test, *P* < .05).

**Figure 7:**
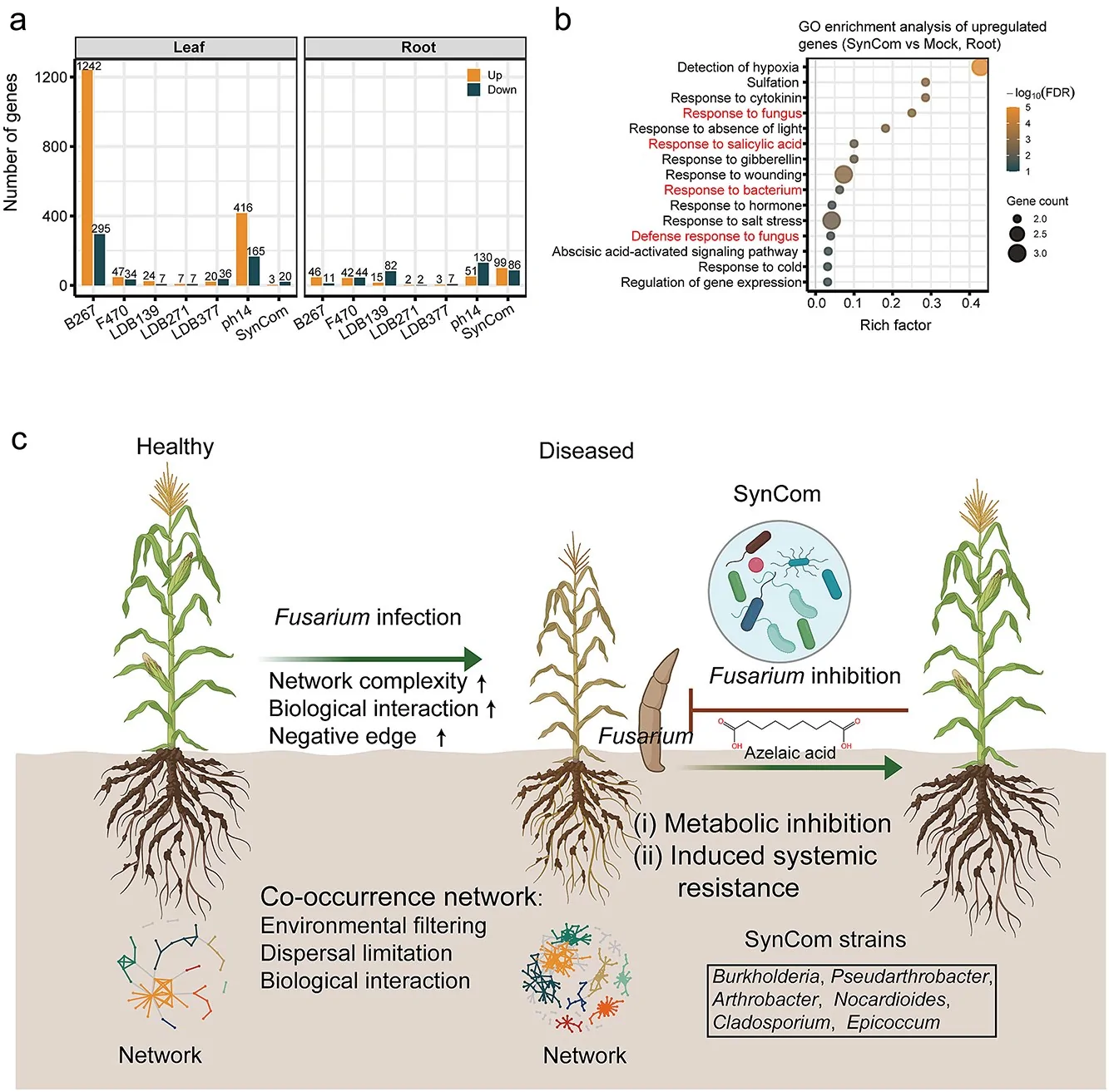
Impact of the SynCom on the maize transcriptome and a schematic model of potential interaction under pathogen stress. (a) Number of differentially expressed genes in maize roots and leaves in response to inoculation with individual strains and the SynCom (FDR adjusted *P* < .05). (b) GO enrichment analysis of upregulated genes in maize roots inoculated with the SynCom (FDR adjusted *P* < .05). (c) A conceptual model depicting the potential interaction network within the maize microbiome and the disease-resistance mechanism mediated by the SynCom under pathogen stress.

## Discussion

An increasing number of studies utilize amplicon sequencing data to construct microbial co-occurrence networks for inferring potential species interactions [6, 37]. However, accumulating evidence underscores significant limitations in interpreting these networks [16, 38, 39], with prominent studies cautioning that co-occurrence patterns alone cannot serve as definitive evidence for ecological interactions [40]. Our study reinforces this critical perspective. The major finding of this study is that ecological refinement of microbial co-occurrence networks improved their ability to identify microbiota with experimentally validated disease-suppressive functions. Rather than treating significant co-occurrence as evidence of biological interaction, we explicitly evaluated the contribution of environmental filtering and dispersal limitation to pairwise microbial associations in a nationwide maize microbiome dataset. More than half of the associations in the original networks were attributable to these processes, demonstrating that large-scale co-occurrence networks can contain substantial ecological structure unrelated to direct biotic interactions.

Microbial network complexity is intrinsically linked to community functionality [11]. In our study, pathogen infection significantly increased the complexity of maize-associated microbial communities and the prevalence of potential negative interactions across all compartments except seeds. This pattern may result from multiple non-mutually exclusive processes, such as intensified microbial competition for limited resources and direct antagonistic inhibition of the pathogen by members of the microbiome [41, 42]. This observation aligns with previous reports in tomato and almond pathosystems, where elevated pathogen pressure increased the frequency of negative edges within microbial co-occurrence networks [43–45]. The observed increase in network complexity aligns with the ecological speculation that plants under biotic stress may modify rhizosphere chemistry to recruit beneficial microorganisms [46]. However, our correlative data do not provide direct evidence for this speculation or other mechanistic claims. Future research combining root exudate profiling, microbial community manipulation, and targeted genetic validation will be required to uncover the causal mechanisms driving the observed restructuring of the microbial network. Seeds, as reproductive organs, exhibit inconsistent microbial network patterns compared with other niches, which may reflect distinct microbiome assembly processes. This observation is consistent with a previous study [47]. Beyond network complexity, we identified distinct phylogenetic patterns in microbial interactions. Potential positive interactions were prevalent among closely related taxa, likely reflecting shared ecological niches and synchronized stress responses [48]. Conversely, phylogenetically distant taxa may be more likely to possess divergent secondary metabolite clusters; this metabolic differentiation may facilitate chemical antagonism, thereby driving the enhanced potential negative interactions observed [49]. However, phylogeny-based inferences still require further validation through metabolic experimentation.

Our investigation revealed contrasting ecological strategies between bacteria and fungi in response to pathogen stress. This divergence may be explained by distinct ecological roles and response mechanisms between the two kingdoms. Previous research indicates that plants under stress conditions secrete a more diverse array of organic compounds, potentially supporting more complex bacterial communities [50]. Furthermore, certain bacterial taxa may directly benefit from pathogen-induced plant tissue degradation, leading to enhanced abundance and metabolic activity [51–52]. In contrast, the structural simplification observed in fungal networks may reflect intensified competitive interactions between fungi and *Fusarium* pathogens, particularly through interference competition for specialized niches or resources. This kingdom-specific response pattern underscores the necessity of separately analyzing bacterial and fungal networks when investigating microbiome responses to biotic stresses. Our investigation revealed that pathogen infection significantly increased the numbers of microbial taxa exhibiting potential negative interactions with *Fusarium* species, with this enrichment being most pronounced in the rhizosphere. This finding suggests that taxa negatively associated with *Fusarium* pathogens represent a valuable reservoir for screening natural biocontrol agents [53–55]. A considerable proportion of taxa demonstrating significant potential negative interactions with *Fusarium* represent uncultivated and taxonomically uncharacterized microbial species. These unexplored microbial resources may possess previously uncharacterized mechanisms of pathogen suppression warranting further investigation.

A growing body of research demonstrates that mining microbial network data for key disease-suppressive taxa and constructing functional synthetic communities is a promising strategy [37, 53, 56, 57]. Our results further substantiate this, showing that the refined network-derived SynCom exhibited superior biocontrol efficacy against maize stalk rot in non-sterile soil compared to SynCom derived from the unrefined network or assembled randomly. These results indicate that network refinement can reduce environmentally structured associations and improve the identification of candidate disease-suppressive taxa. Our filtering of environmentally dependent taxa may exclude taxa that confer disease resistance locally. These locally functional taxa represent an important resource for context-specific disease suppression and will be a key focus of future research. The resulting SynCom, validated in non-sterile soil, represents a core consortium of antagonists. For future agricultural applications, this core consortium could be augmented with region-specific strains identified through local network refinements to maximize efficacy across heterogeneous agroecosystems [56, 57]. An important limitation of our approach is the taxonomic resolution imposed by marker-gene amplicon sequencing. The V5–V6 region of the bacterial 16S rRNA gene and the fungal ITS2 region can resolve distinct sequence variants, but generally do not provide reliable species- or strain-level identification. The isolates used in this study should therefore be regarded as culturable representatives corresponding to the target amplicon-defined taxa. Nevertheless, this approach provides a practical and efficient screening strategy for rapidly identifying isolates with high sequence similarity to target taxa and for constructing function-oriented synthetic communities. Future studies integrating metagenomic sequencing with whole-genome sequencing of individual isolates will be required to achieve more precise microbiome manipulation.

SynComs enhance plant growth and disease resistance through multiple possible mechanisms, including niche competition, production of antagonistic compounds, and modulation of host immunity [5, 43, 58, 59]. Mechanistic investigations revealed that the SynCom likely functions via direct antagonism and indirect induction of plant immunity. Key antimicrobial metabolites identified in this study, including azelaic acid and fragin, have previously been reported to inhibit diverse fungal pathogens [60, 61]. The antifungal activity of boholamide A against *Fusarium* remains poorly characterized. Transcriptomic profiling further showed that SynCom inoculation induced the expression of salicylic acid-mediated defense pathway genes in the roots, suggesting activation of root defense pathways that may contribute to enhanced disease resistance [62]. Collectively, these findings demonstrate the potential of rationally designed SynCom to enhance plant health and disease resistance reliably and efficiently through synergistic, multi-mechanistic actions. Future validation of the SynCom’s disease-suppressive efficacy in field trials and across heterogeneous environmental contexts is warranted. Whereas our validation experiments have focused on the rhizosphere, other niches, including the root, stem, and seed, also harbor microbial resources that warrant further investigation, particularly given that the stem tissue constitutes the primary compartment of infection for maize stalk rot. A further limitation of the current network analysis is its reliance on pairwise correlations, whereas higher-order interactions (non-additive effects emerging from multi-species interactions) may also play critical roles in community dynamics and function [63, 64]. Future research should develop more sophisticated models and algorithms to elucidate these complex ecological interactions.

In summary, our large-scale field study demonstrates substantial contributions of environmental selection and dispersal limitation to the structure of maize microbial co-occurrence networks. By constructing ecologically refined interaction networks, we show that pathogen infection alters the structure and topological complexity of the maize microbiome. This structural reorganization was characterized by an increased prevalence of potential negative interactions and the enrichment of specific microbial taxa exhibiting potential negative interactions with *Fusarium* pathogens. The SynCom assembled from network-identified *Fusarium*-antagonistic taxa significantly reduced the maize stalk rot disease index. Collectively, our findings support an integrated analytical–experimental workflow for investigating multi-kingdom plant–pathogen–microbe interactions and offer an ecologically informed strategy for managing this globally widespread maize disease.

## Supplementary Material

Supplementary_material_wrag243

## Data Availability

The raw sequence data reported in this paper have been deposited in the Genome Sequence Archive at the National Genomics Data Center, China National Center for Bioinformation/Beijing Institute of Genomics, Chinese Academy of Sciences (GSA: CRA017629, CRA017631), and are publicly accessible at https://ngdc.cncb.ac.cn/gsa. All other data are available from the corresponding author upon reasonable request. The data visualization code can be found in https://github.com/lidas666/Network.
