# Supplementary material for: Network-guided synthesis of antagonistic microbiota for suppressing maize stalk rot disease
Source: ISME J. 2026 Oct 6;20(1):wrag243. doi: 10.1093/ismejo/wrag243 (PMC13712302; doi:10.1093/ismejo/wrag243)
Supplement: Supplementary file 1 [file supplementary_material_wrag243.zip › Supplementary information_final.docx]

**Supplementary information for**

**Network-guided synthesis of antagonistic microbiota for suppressing maize stalk rot disease**

Da Li^1,2^, Zishuo Qu^1,2^, Wenzhe Feng^1^, Xin Zhou^1*^, and Lei Cai^1,2*^

^1^State Key Laboratory of Microbial Diversity and Innovative Utilization, Institute of Microbiology, Chinese Academy of Sciences, Beijing 100101, P. R. China

^2^College of Life Science, University of Chinese Academy of Sciences, Beijing 100049, P. R. China.

*Corresponding author: Xin Zhou, email: [zhouxin@im.ac.cn](mailto:zhouxin5518@163.com); Lei Cai, email: [cail@im.ac.cn](mailto:cail@im.ac.cn;)

**Contents of Supplementary Information**

**Supplementary Figures**

**Supplementary Fig. 1 Impact of *Fusarium* stalk rot infection on maize bacterial-fungal co-occurrence networks.**

**Supplementary Fig. 2 Influence of climatic, geographic, and edaphic factors on maize-associated microbiota.**

**Supplementary Fig. 3 Effects of environmental and geographical factors on pairwise associations in the microbial co-occurrence networks of healthy and diseased maize**

**Supplementary Fig. 4 Phylogenetic analysis of associations in the maize microbiome co-occurrence network.**

**Supplementary Fig. 5 Impact of *Fusarium* stalk rot infection on microbial community potential interaction networks in maize.**

**Supplementary Fig. 6 Impact of *Fusarium* stalk rot infection on bacterial and fungal co-occurrence networks in maize.**

**Supplementary Fig. 7 Impact of *Fusarium* stalk rot infection on bacterial and fungal potential interaction networks in maize.**

**Supplementary Fig. 8 Effect of maize stalk rot on the abundance of *Fusarium* ZOTUs in different maize tissues.**

**Supplementary Fig. 9 Phylogenetic analysis of *Fusarium* ZOTUs based on ITS sequences.**

**Supplementary Fig. 10 Overlap of taxa negatively correlated with *Fusarium* between the co-occurrence network at a single sampling site and the refined network in the rhizosphere of diseased maize.**

**Supplementary Fig. 11 Overlap of taxa negatively correlated with *Fusarium* between the SPIEC-EASI derived co-occurrence network and the refined network in the rhizosphere of diseased maize.**

**Supplementary Fig. 12 Effects of network refinement on microbial taxa negatively correlated with *Fusarium* in maize stem.**

**Supplementary Fig. 13 Impact of SynCom on disease index of maize stalk rot.**

**Supplementary Fig. 14 Effects of individual bacterial strains on maize stalk rot disease index and the fungal pathogen.**

**Supplementary Fig. 15 Effects of individual strains from the random SynCom, unrefined network SynCom, and refined network SynCom on maize stalk rot disease index.**

**Supplementary Fig. 16 Temporal dynamics of the relative abundance of individual members within the SynComs following inoculation.**

**Supplementary Fig. 17 Shared and unique metabolites between individual strains and the SynCom.**

**Supplementary Fig. 18 GO enrichment analysis of maize transcriptomes inoculated with SynComs.**

**Supplementary Fig. 19 Differential enrichment analysis of maize transcriptomes inoculated with single strains and a SynCom.**

**Supplementary Fig. 20 GO analysis of maize transcriptomes inoculated with single strains and a SynCom.**

**Supplementary Tables**

**Supplementary Table 1** Topological properties of the co-occurrence network under healthy or diseased maize in different compartments.

**Supplementary Table 2** Topological properties of the co-occurrence random network under healthy or diseased maize in different compartments.

**Supplementary Table 3** Drivers of cross-kingdom co-occurrence association pairs in the maize microbiome.

**Supplementary Table 4** Proportion of environmental factors driving the cross-kingdom co-occurrence network in the maize microbiome.

**Supplementary Table 5** The proportion of ecological processes that drive the association of microbial networks

**Supplementary Table 6** Topological properties of the potential interaction networks under healthy or diseased maize in different compartments.

**Supplementary Table 7** Topological properties of the potential random interaction networks under healthy or diseased maize in different compartments.

**Supplementary Table 8** Taxa negatively correlated with *Fusarium*.

**Supplementary Table 9** Comparison of Fusarium-negatively correlated taxa identified from single-site networks and the refined network.

**Supplementary Table 10** Overlapping taxa identified as Fusarium-negatively correlated in both single-site networks and the refined network.

**Supplementary Table 11** Overlapping taxa identified as Fusarium-negatively correlated in both SPIEC-EASI and the refined network.

**Supplementary Table 12** Information on rhizosphere-isolated strains.

**Supplementary Table 13** SynCom strain information.

**Supplementary Table 14** Potential associations between metabolites and antifungal capacity based on random forest analysis.

**Supplementary Table 15** Differentially expressed genes in maize transcriptomes following inoculation with individual strains and the SynCom.

**Supplementary Table 16** GO enrichment analysis in maize transcriptomes following inoculation with the SynComs.

**Supplementary Fig. 1 Impact of *Fusarium* stalk rot infection on maize bacterial-fungal co-occurrence networks.** (A) Visualization of bacterial-fungal co-occurrence networks across distinct maize niches under pathogen stress. The top eight major modules are highlighted in color, with remaining modules shown in gray. (B) Topological properties of bacterial-fungal co-occurrence networks under pathogen stress.

**Supplementary Fig. 2 Influence of climatic, geographic, and edaphic factors on maize-associated microbiota.** (A) Canonical correspondence analysis (CCA) of microbial communities across maize compartments. (B) Variation partitioning analysis (VPA) quantifying contributions of environmental drivers to microbial community variation. Asterisks denote statistical significance: **P* < 0.05, ***P* < 0.01, ****P* < 0.001; ns: not significant.

**Supplementary Fig. 3 Effects of environmental and geographical factors on pairwise associations in the microbial co-occurrence networks of healthy and diseased maize.** UTM_X and UTM_Y represent the easting and northing distances, respectively, in the Universal Transverse Mercator coordinate system. Significance was defined as *P* < 0.05.

**Supplementary Fig. 4 Phylogenetic analysis of associations in the maize microbiome co-occurrence network.** (A) Phylogenetic distances between taxa linked by co-occurrence relationships governed by environmental selection, dispersal limitation, and biological interactions. The combined bacterial–fungal phylogenetic tree was constructed using the maximum-likelihood method. (B) Differences in phylogenetic distance for positive associations in the microbiome networks of healthy and diseased maize. (C) Differences in phylogenetic distance for negative associations in the microbiome networks of healthy versus diseased maize. Differences were tested using the Wilcoxon rank-sum test.

**Supplementary Fig. 5 Impact of *Fusarium* stalk rot infection on microbial community potential interaction networks in maize.** L: Number of edges; Pos: Number of positive correlations; Neg: Number of negative correlations; n: Number of nodes; APL: Average path length; Con: Connectance; AK: Average degree; RM: Relative modularity; ACC: Average clustering coefficient; N. clusters: Number of clusters; Betw: Betweenness centrality; Clos: Closeness centrality.

**Supplementary Fig. 6 Impact of *Fusarium* stalk rot infection on bacterial and fungal co-occurrence networks in maize.** (A) Topological properties of bacterial co-occurrence networks across distinct maize niches under pathogen stress. (B) Topological properties of fungal co-occurrence networks across distinct maize niches under pathogen stress.

**Supplementary Fig. 7 Impact of *Fusarium* stalk rot infection on bacterial and fungal potential interaction networks in maize.** (A) Topological properties of bacterial potential interaction networks across distinct maize niches under pathogen stress. (B) Topological properties of fungal potential interaction networks across distinct maize niches under pathogen stress.

**Supplementary Fig. 8 Effect of maize stalk rot on the abundance of *Fusarium* ZOTUs in different maize tissues.** Differences were tested using the Wilcoxon rank-sum test.

**Supplementary Fig. 9 Phylogenetic analysis of *Fusarium* ZOTUs based on ITS sequences.** The phylogenetic tree was constructed using the maximum likelihood method.

**Supplementary Fig. 10 Overlap of taxa negatively correlated with *Fusarium* between the co-occurrence network at a single sampling site and the refined network in the rhizosphere of diseased maize.** (A) Venn diagram illustrating the number of shared and unique taxa negatively correlated with *Fusarium* in the co-occurrence network at a single sampling site versus the refined network. (B) Proportion of taxa shared between the co-occurrence network at a single sampling site and the refined network that are negatively correlated with *Fusarium*.

**Supplementary Fig. 11 Overlap of taxa negatively correlated with Fusarium between the SPIEC-EASI derived co-occurrence network and the refined network in the rhizosphere of diseased maize.**

**Supplementary Fig. 12 Effects of network refinement on microbial taxa negatively correlated with *Fusarium* in maize stem.** (A) Comparison of *Fusarium*-negatively correlated taxa identified from co-occurrence networks before (orange) and after (green) filtering out taxa influenced by environmental and geographic variation. Only taxa with significant differences (*P* < 0.05) in random forest models are shown. (B) Importance of *Fusarium*-negatively associated taxa in explaining variation in *Fusarium* relative abundance variation before and after removing environment and/or geography-dependent taxa. (C-E) Distance-decay curves showing Bray–Curtis similarity against climatic distances (C), geographic distances (D) and edaphic distances (E). Asterisks denote statistical significance: ****P* < 0.001.

**Supplementary Fig. 13 Impact of SynCom on disease index of maize stalk rot.** Fv: *F. verticillioides* LD38; Fg: *F. graminearum* LD190.

**Supplementary Fig. 14 Effects of individual bacterial strains on maize stalk rot disease index and the fungal pathogen.** (A-F) Effects of individual bacterial strains on the maize stalk rot disease index: strains from the random community (A, D), unrefined network SynCom (B, E), and refined network SynCom (C, F) (scale bar = 10 cm). (G-L) Effects of individual bacterial strains on the growth of *F. verticillioides* LD38: strains from the random community (G, J), unrefined network SynCom (H, K), and refined network SynCom (I, L) (scale bar = 1 cm). Different lowercase letters indicate significant differences between treatments (Kruskal-Wallis H test, *P* < 0.05).

**Supplementary Fig. 15 Effects of individual strains from the random SynCom, unrefined network SynCom, and refined network SynCom on maize stalk rot disease index.** Different lowercase letters indicate significant differences between treatments (Kruskal-Wallis test, *P* < 0.05).

**Supplementary Fig. 16 Temporal dynamics of the relative abundance of individual members within the SynComs following inoculation.** Line plots illustrate the temporal trajectories of the relative abundance of each strain in the synthetic community across three successive time points (10, 20, and 30 dpi). Each line represents one individual strain, with points denoting biological replicates and error bars indicating the standard error of the mean.

**Supplementary Fig. 17** **Shared and unique metabolites between individual strains and the SynCom.**

**Supplementary Fig. 18 GO enrichment analysis of maize transcriptomes inoculated with SynComs.**

**Supplementary Fig. 19 Differential enrichment analysis of maize transcriptomes inoculated with single strains and a SynCom.** (A) Differential enrichment analysis of leaf transcriptomes. (B) Differential enrichment analysis of root transcriptomes.

**Supplementary Fig. 20 GO analysis of maize transcriptomes inoculated with single strains and a SynCom.** (A) GO analysis of leaf transcriptomes. (B) GO analysis of root transcriptomes.
